# Estimates of the excess cost burden of Ehlers-Danlos syndromes: a United States MarketScan® claims database analysis

**DOI:** 10.3389/fpubh.2024.1365712

**Published:** 2024-07-03

**Authors:** Jane R. Schubart, Eric W. Schaefer, Dacre R. T. Knight, Susan E. Mills, Clair A. Francomano

**Affiliations:** ^1^Department of Surgery, Penn State College of Medicine, Hershey, PA, United States; ^2^Department of Public Health Sciences, Penn State College of Medicine, Hershey, PA, United States; ^3^Division of General Internal Medicine, Mayo Clinic, Jacksonville, FL, United States; ^4^Department of Medical and Molecular Genetics, Indiana University School of Medicine, Indianapolis, IN, United States

**Keywords:** Ehlers-Danlos syndromes, hypermobility, economic, cost, MarketScan, commercial health insurance, health policy

## Abstract

**Introduction:**

Patients with Ehlers-Danlos syndromes (EDS) and hypermobility spectrum disorders (HSD) have significant health challenges that are well-documented, however their impact in terms of cost is not known. Our research objective was to examine the cost burden of EDS and HSD in the United States. We focused this analysis on those with commercial insurance plans.

**Methods:**

We queried the MarketScan® database for year 2021 for claims that contained an ICD-10 diagnosis code for EDS or hypermobility. Excess costs for patients in the EDS and HSD cohorts were determined by matching each patient to one patient in the database that did not have a claim for EDS or HSD and comparing total costs for the calendar year. We determined whether patients had claims for selected comorbid conditions likely to impact costs during the calendar year.

**Results:**

Sample sizes were 5,113 for adult (age ≥ 18) patients with EDS, 4,880 for adult patients with HSD, 1,059 for child (age 5–17) patients with EDS, and 2,427 for child patients with HSD. The mean excess costs were $21,100 for adult EDS patients, $11,600 for adult HSD patients, $17,000 for child EDS patients, and $11,000 for child HSD patients. EDS and HSD cohorts, both adults and children, with any of the comorbidities had greater healthcare costs. The largest difference was found in the EDS cohort with gastrointestinal comorbid conditions, with more than double the costs for adults.

**Discussion:**

We found that patients in the MarketScan database, adults and children, who had EDS or HSD had substantially higher associated excess healthcare costs than patients without EDS or HSD when considering age, sex, geographic location, and comorbidities. These disproportionate healthcare costs in this population have health policy and economic implications, including the need for rapid diagnosis, access to treatment, and accelerated research to advance treatments.

## 1 Introduction

The Ehlers-Danlos syndromes (EDS) are heritable connective tissue disorders characterized by varying degrees of joint hypermobility and instability, skin hyperextensibility and scarring, and other consequences of tissue fragility such as vascular and visceral rupture (1). Most types of EDS are rare; however, the hypermobile type (hEDS) (2) is more common (3). Hypermobility spectrum disorder (HSD) is a related diagnosis with hypermobility causing musculoskeletal pain and injury (4, 5). Patients with symptomatic joint hypermobility who do not meet the 2017 diagnostic criteria for hEDS are often assigned the diagnosis of HSD. Vascular type is the most severe type with features that may include arterial rupture at a young age, unexplained sigmoid colon perforation, uterine rupture, and other life-threatening events (1).

Symptoms of EDS typically begin in childhood and adolescence, worsen throughout young adulthood (most often in women in the peri-pubertal years), and can lead to chronic complex multi-system concerns, requiring multidisciplinary care by medical, surgical, and therapy specialties (2). Some comorbid conditions of EDS are not included in the formal diagnostic criteria (2), including sleep disturbance, fatigue, postural orthostatic tachycardia, disorders of the gut-brain interaction, dysautonomia, anxiety, and depression. These other systemic manifestations may be more debilitating than the joint symptoms (1). Patients have highly variable symptoms and treatment needs; thus, treatment is typically managed by multiple providers. There is a growing body of literature documenting the significant health challenges of EDS and HSD (2, 6–8). Many individuals will endure years without proper diagnoses and needed treatments. After diagnosis, it is common for patients to seek treatment for multiple issues at once, for extended periods of time (8). Treatments are individualized and focus on managing a wide range of symptoms and comorbidities that may include physical therapy, exercise, lifestyle modification, medication and when needed, surgical intervention. For this reason, published studies establishing the “value” of treatments for EDS and HSD are lacking.

Our research objective was to examine the cost burden of EDS and HSD in the United States. We focused this analysis on the population with commercial insurance plans for two reasons. First, over half of the total U.S. population receives health insurance through commercial plans that are offered by employers or purchased by individuals (9) and spending by commercial health insurers on hospital and physician services has grown faster than spending by the Medicare fee-for-service program. Second, the MarketScan database (10) provides a large sample across all 50 states of Americans with employer-provided health insurance and captures complete episodes of care.

## 2 Materials and methods

### 2.1 Data source

Data were extracted from the Merative™ MarketScan® Commercial Claims and Encounters database (10). MarketScan contains reimbursed claims data on over 273 million unique patients since 1995. The database includes employees, dependents, and retirees receiving coverage annually under private insurance plans. No Medicaid or Medicare data are included. The database contains information regarding inpatient and outpatient claims, and prescription drug claims. It is one of the largest and longest running proprietary claims databases used for healthcare research in the U.S.

### 2.2 Construction of cohorts

We searched all inpatient and outpatient claims in the MarketScan database for the calendar year 2021 for claims that contained an International Classification of Diseases, Tenth Revision, Clinical Modification (ICD-10) diagnosis code for EDS (Q79.6, Q79.60, Q79.61, Q79.62, Q79.63, Q79.69). These are the only codes for EDS. Because there are no current ICD-10 codes for HSD, we used the code for hypermobility syndrome (M35.7) as a surrogate. Other inclusion/exclusion criteria were age ≥ 5 years old and continuously enrolled in an insurance plan for the calendar year 2021 with prescription drug coverage included in the individual’s insurance plan. We constructed two separate cohorts: (1) patients with a diagnosis code for EDS (with or without coding for hypermobility); (2) patients with a diagnosis code for hypermobility only (no EDS diagnosis code).Additionally, each cohort was separated into adult (age ≥ 18) and child (age ≥ 5 to <18) cohorts.

All inpatient and outpatient claims that occurred during the calendar year 2021 were searched for claims with specific ICD-10 diagnosis codes that indicated chronic comorbidities. From these claims, we calculated a recent edition of the Charlson Comorbidity Index (CCI) score, which is particularly useful for claims-based research purposes (11).

### 2.3 Matched comparison group

We estimated the excess costs for patients in our EDS and HSD cohorts by matching each patient to one patient in the database that did not have a claim for EDS or HSD during 2021 and were continuously enrolled with prescription drug claim capture for the calendar year. A total of 14,263,020 patients met these criteria and formed the set of possible matches for our EDS and HSD patients. We matched patients on age (+/− 2 years), sex, Census division, and CCI score (+/− 1 point) using a greedy matching algorithm (12). 88.8% of the matches in the EDS cohort and 91.7% in the HSD cohort were exact. We compared mean total healthcare costs in 2021 between groups.

### 2.4 Variables

#### 2.4.1 Healthcare costs

Total healthcare costs were calculated for the calendar year 2021. All costs from inpatient claims, outpatient claims, and pharmaceutical claims that occurred during the year were summed to create the total healthcare costs for a patient in 2021. The value represents the amount eligible for payment under the medical plan terms after applying rules such as discounts, but before applying coordination of benefits, copayments, and deductibles. We also calculated the total healthcare costs for each type of claim (inpatient, outpatient, emergency department, and pharmaceutical). For a small proportion of patients (<10 for each cohort; <0.1%), total healthcare costs were negative. Negative costs indicate reimbursement or other adjustments to a previous error in billing. We imputed a value of $0 for these patients.

#### 2.4.2 Ehlers-Danlos claims

We identified ED claims using the sub-service category code in the MarketScan database. A code with the last two digits of ‘20’ indicated an ED visit.

#### 2.4.3 Ehlers-Danlos syndromes/HSD comorbidities

We determined whether patients had claims for selected comorbid conditions during the calendar year 2021. We used these specific comorbidities because these are the most frequently occurring comorbidities affecting patients with EDS/HSD that are likely to impact healthcare costs. Patients were classified as having the comorbidity if any claim was found with the respective code during the calendar year (13–31).The comorbidities were determined using the following ICD-10 diagnosis codes:

- Dysautonomia (POTS): G90.8, G90.9, I49.8, G90.A, I95.1, R55, R00.2
- Mast cell disorders: D89.40-D89.44, D89.49
- Gastrointestinal: K31.84, K58, K58.1, K21
- Depression/anxiety: F32.x, F33.x, F41.x, F43.x
- Musculoskeletal: S13.x, S23.x, S33.x, S43.x, S53.x, S63.x, S73.x, S83.x, S93.x, M81.x, M80.x, M26.6x
- Neurological: G93.5, Q07.0x, G95.89, G06.9, G54.0, G43.x, M24.80
- Other: R53.82, F84.0, M79.7, G47.x, G90.50x, N94.89, I87.2, I77.9x, I87.1, I77.4, N30.1x, N80.x, I34.0, I34.1, I71.x

### 2.5 Statistical analysis

Excess costs for patients in the EDS and HSD cohorts were determined by matching each patient to one patient in the database that did not have a claim for EDS or HSD and comparing total costs for the calendar year. We reported descriptive statistics (mean, standard deviation [SD], median, interquartile range [IQR], and range) for total healthcare costs. Cost estimates were rounded to the nearest $100.

For patients with EDS, we examined costs for patients who had a diagnosis code for vascular type (ICD-10: Q79.63) compared to those without a vascular diagnosis coded. Separately, we examined costs for patients with EDS who had a diagnosis code for hypermobile type (ICD-10: Q79.62) and compared to HSD patients.

To estimate excess costs, we calculated the difference in mean total healthcare costs between the cohorts and their matched comparison group. We used bootstrapping to estimate 95% CIs. Specifically, we used 1,000 resamples and the percentile method to obtain CIs.

To model costs with respect to EDS comorbidities, we used generalized linear regression models. For these models, we assumed a Gamma distribution and used a log link. The Gamma distribution was selected because costs exhibited substantial skewness, with some patients having very large costs. The models contained indicators for each comorbidity, age, sex, and CCI score with age and CCI score modeled linearly. Results from the models were reported in two ways. First, we reported cost ratios (CRs) and corresponding 95% confidence intervals (CIs). The cost ratios represent the multiplicative costs for each comorbidity. Second, we reported marginal differences in costs on the dollars scale. To estimate marginal costs, we set all other variables in the model to the most common value or the median as a reference. Specifically, marginal estimates were calculated for each comorbidity after assuming no other comorbidities, age of 34 (adult cohorts) or 14 (child cohorts), female sex, and CCI score of 0 as the reference, which we refer to below as the “reference patient.”

## 3 Results

### 3.1 Patient characteristics

Sample sizes for each cohort were 5,113 and 1,059 for adult and child EDS cohorts, respectively, and 4,880 and 2,427 for adult and child HSD cohorts, respectively.

**Figure 1:**
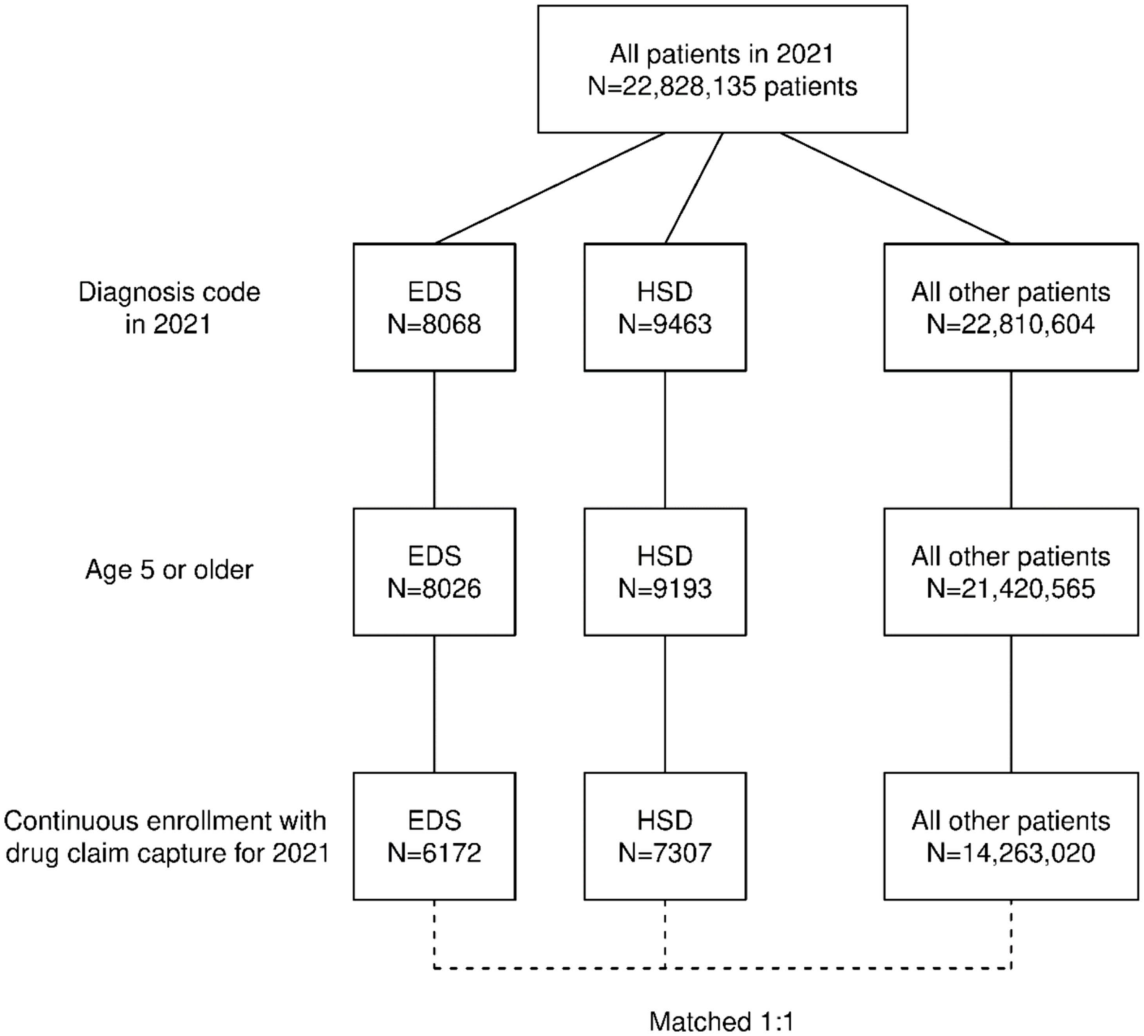
Construction of the cohort.

A flowchart depicting how these cohorts were constructed is shown in Figure 1. In the calendar year 2021, the database contained a total of 22,828,135 patients. Diagnosis codes for EDS and HSD were found for 8,068 and 9,463 patients, respectively (<0.1% for each). When restricting the cohorts to patients age ≥ 5 years old, 0.5% of the EDS cohort and 2.9% of the HSD cohort were excluded. Then, an additional 23.1 and 20.5% for EDS and HSD cohorts, respectively, were excluded because they did not have continuous enrollment with prescription drug claim capture for the entire calendar year. In total, 76.5% of EDS patients and 77.2% of HSD patients were retained for the analysis. In contrast, for all other patients in the database, only 62.5% were retained after applying the same inclusion/exclusion criteria. The larger percentage excluded was primarily due to the continuous enrollment exclusion, which indicated that patients with EDS and HSD were more likely to have commercial insurance for the entire calendar year.

Table 1 shows characteristics and comorbidities for the EDS and HSD cohorts.

**Table 1:** Patient characteristics.

	EDS Adult	EDS Child	HSD Adult	HSD Child
*N*	5,113	1,059	4,880	2,427
Age, years				
Mean (SD)	35.4 (12.7)	13.5 (3.1)	37.5 (13.2)	12.7 (3.4)
Median	34	14	37	14
IQR	23, 45	12, 16	25, 48	10, 16
Range	18, 64	5, 17	18, 64	5, 17
Sex, *n* (%)				
Male	603 (11.8%)	362 (34.2%)	630 (12.9%)	751 (30.9%)
Female	4,510 (88.2%)	697 (65.8%)	4,250 (87.1%)	1,676 (69.1%)
CCI Score				
Mean (SD)	1.1 (2.1)	0.4 (1.2)	0.9 (1.9)	0.4 (1.2)
Median	0.0	0.0	0.00	0.0
IQR	0, 1	0, 1	0, 1	0, 0
Range	0, 15	0, 10	0, 13	0, 10
CCI Score, *n* (%)				
0	2,892 (56.6%)	789 (74.5%)	2,939 (60.2%)	1,956 (80.6%0)
1	1,250 (24.4%)	218 (20.6%)	1,166 (23.9%)	360 (14.8%)
2	340 (6.6%)	20 (1.9%)	307 (6.3%)	34 (1.4%)
3	103 (2.0%)	5 (0.5%)	79 (1.6%)	7 (0.3%)
≥ 4	528 (10.3%)	27 (2.5%)	389 (8.0%)	70 (2.9%)
Comorbidities, *n*(%)				
Dysautonomia (POTS)	2,003 (39.2%)	301 (28.4%)	917 (18.8%)	323 (13.3%)
Mast cell disorders	442 (8.6%)	48 (4.5%)	119 (2.4%)	26 (1.1%)
Gastrointestinal	401 (7.8%)	53 (5.0%)	138 (2.8%)	33 (1.4%)
Depression/anxiety	2,916 (57.0%)	494 (46.6%)	2,373 (48.6%)	858 (35.4%)
Musculoskeletal	1,071 (20.9%)	200 (18.9%)	1,031 (21.1%)	468 (19.3%)
Neurological	1,720 (33.6%)	240 (22.7%)	1,077 (22.1%)	411 (16.9%)
Other	2,315 (45.3%)	297 (28.0%)	1,763 (36.1%)	448 (18.5%)

IQR: interquartile range.

In the EDS cohort, 87.3% of adults and 72.8% of children had one or more EDS/HSD comorbid conditions, compared to 75.6% of adults and 60.7% of children in the hypermobility cohort. In the EDS cohort, 36.0% of adults and 21.4% of children had 3 or more EDS/HSD comorbid conditions, compared to 21.4% of adults and 11.9% of children in the hypermobility cohort.

Patient characteristics for those included in the sample were generally similar to those who were excluded. For all EDS patients (adults and child cohorts) who were excluded, the median age was 29 (compared to 30 for those included), 85.3% were female (compared to 84.4%) and 62.5% had a CCI score of 0 (compared to 59.6%). Similarly, for all HSD patients (adult and child cohorts) who were excluded, the median age was 27 (compared to 25), 82.4% were female (compared to 81.1%), and 71.3% had CCI score of 0 (compared to 67.0%).

Table 2 shows diagnoses found in claims during the year for the EDS cohort. These diagnoses are not mutually exclusive because patients could have claims for any of the diagnoses during the calendar year.

**Table 2:** Diagnoses for EDS cohort.

	Child (*N* = 1,059)	Adult (*N* = 5,113)
Diagnosis code, *n* (%)		
Q79.60 (Unspecified)	831 (78.5%)	4,158 (81.3%)
Q79.61 (Classical)	47 (4.4%)	256 (5.0%)
Q79.62 (Hypermobile)	357 (33.7%)	1,613 (31.5%)
Q79.63 (Vascular)	12 (1.1%)	87 (1.7%)
Q79.69 (Other)	67 (6.3%)	238 (4.7%)

### 3.2 Total healthcare costs

Table 3 shows the total healthcare costs overall, and by each claim source (outpatient, inpatient, pharmaceutical, and Emergency Department). By definition, all of the cohorts had outpatient claims. 13.3% of adults with EDS and 9.5% of children had inpatient claims, nearly two-fold compared to the HSD cohorts. The vast majority had pharmaceutical claims which is not surprising given the multi-system manifestations of the conditions. Approximately one-third of those with EDS and one quarter with HSD had ED claims.

**Table 3:** Healthcare costs by cohort and type.

	EDS Adult	EDS Child	HSD Adult	HSD Child
Total healthcare costs	N = 5,113	1,059	N = 4,880	N = 2,427
Mean (SD)	$32,800 ($63,000)	$21,600 ($45,500)	$22,100 ($37,700)	$15,000 ($37,200)
Median	$13,800	$7,100	$9,500	$4,600
IQR	$5,500, $34,000	$2,700, $19,800	$3,800, $24,400	$1,800, $11,700
Range	$0, $1,135,700	$0, $469,600	$0, $749,300	$0, $718,800
Outpatient claims	N = 5,113	N = 1,059	N = 4,880	N = 2,427
%	100.0	100.0	100.0	100.0
Mean (SD)	$19,600 ($32,300)	$13,600 ($24,600)	13,900 (24,500)	$9,600 ($19,700)
Median	$9,300	$5,800	6,800	$3,900
IQR	$3,900, $21,200	$2,300, $14,200	2,900, 15,600	$1,600, $9,700
Range	$0, $578,900	$0, $321,400	0, 748,400	$0, $324,800
Inpatient claims	N = 680	N = 101	N = 273	N = 125
%	13.3	9.5	5.6	5.2
Mean (SD)	$47,900 ($76,800)	$43,200 ($61,800)	$37,500 ($39,600)	$50,300 ($59,300)
Median	$23,000	$21,400	$22,400	$25,900
IQR	$13,200, $47,000	$11,100, $49,400	$12,400, $45,700	$13,600, $64,100
Range	$0, $754,800	$1,200, $378,400	$100, $227,300	$0, $298,400
Outpatient pharmaceutical claims	N = 4,828	N = 906	N = 4,586	N = 2,002
%	94.4	85.6	94.0	82.5
Mean (SD)	$7,200 ($29,600)	$4,500 ($19,900)	$6,400 ($21,100)	$3,300 ($18,200)
Median	$1,200	$400	$700	$200
IQR	$300, $5,000	$100, $1,800	$200, $3,000	$100, $1,000
Range	$0, $1,061,700	$0, $281,000	$0, $324,100	$0, $358,800
ED claims	N = 1,989	N = 347	N = 1,258	N = 577
%	38.9	32.8	25.8	23.8
Mean (SD)	$7,000 ($12,400)	$4,800 ($7,500)	$5,000 ($7,000)	$4,600 ($12,300)
Median	$3,400	$2,300	$2,900	$2,200
IQR	$1,700, $7,400	$1,100, $5,300	$1,300, $5,700	$1,100, $4,400
Range	$0, $238,800	$0, $55,000	$0, $79,300	$0, $263,200

### 3.3 Vascular type

Cardiovascular complications have been noted in all EDS subtypes due tissue fragility, however, patients with vascular type EDS are at greater risk of life-threatening events including cerebrovascular events, aneurysm, and arterial dissection/rupture. For this reason, we compared vascular type to non-vascular types. For the adult EDS cohort, Table 4 shows total healthcare costs, by each claim source, stratified by whether patients had vascular type of EDS. For the child EDS cohort, only 12 patients had a diagnosis of vascular type, and therefore, we did not conduct this analysis. Mean costs were higher for patients with vascular EDS across all categories except outpatient pharmaceutical claims. This finding is consistent with the potential catastrophic manifestations of this type.

**Table 4:** Healthcare costs for Vascular EDS.

	EDS adult No vascular diagnosis	EDS adult Vascular diagnosis
Total healthcare costs	N = 5,026	N = 87
Mean (SD)	$32,100 ($60,400)	$70,400 ($146,400)
Median	$13,800	$16,700
IQR	$5,600, $33,800	$5,000, $66,400
Range	$0, $1,135,700	$400, $1,029,300
Outpatient claims	N = 5,026	N = 87
Mean (SD)	$19,400 ($31,900)	$31,400 ($50,800)
Median	$9,300	$11,200
IQR	$3,900, $21,100	$3,200, $33,000
Range	$0, $578,900	$200, $259,200
Inpatient claims	N = 757	N = 24
Mean (SD)	$44,600 ($68,300)	$130,700 ($172,900)
Median	$22,400	$75,600
IQR	$12,900, $45,700	$26,100, $172,100
Range	$0, $627,000	$10,600, $754,800
Outpatient pharmaceutical claims	N = 4,743	N = 85
Mean (SD)	$7,300 ($29,800)	$3,900 ($8,500)
Median	$1,200	$500
IQR	$300, $5,000	$100, $2,900
Range	$0, $1,061,700	$0, $49,300
ED claims	N = 1,950	N = 39
Mean (SD)	$6,800 ($11,600)	$16,900 ($31,600)
Median	$3,300	$5,800
IQR	$1,600, $7,300	$2,300, $15,300
Range	$0, $238,800	$100, $134,000

### 3.4 Healthcare costs for hypermobile type EDS compared to HSD

We compared total healthcare costs between patients with hypermobile type EDS (hEDS) and HSD patients. Table 5 shows total healthcare costs for hEDS compared to HSD. Mean costs were higher for patients with hEDS. Costs were also higher across all categories except inpatient claims which were approximately the same (data not shown). This finding is consistent with the clinical observation that individuals with hEDS are likely to be more severe in terms of their clinical presentation than those with HSD due to the coexisting conditions associated with EDS.

**Table 5:** Healthcare costs for hEDS compared to HSD.

	hEDS adult	HSD adult
Total healthcare costs	N = 1,613	N = 4,880
Mean (SD)	$36,300 ($7,200)	$22,100 ($37,700)
Median	$16,400	$9,500
IQR	$7,100, $37,200	$3,800, $24,400
Range	$260, $1,135,700	$0, $749,300
	**hEDS Child**	**HSD Child**
Total healthcare costs	N = 357	N = 2,247
Mean (SD)	$27,200 ($51,700)	$14,900 ($37,200)
Median	$9,100	$4,600
IQR	$4,000, $25,800	$1,800, $11,700
Range	$100, $417,600	$0, $718,300

### 3.5 Estimated cost ratios and marginal differences in costs

Table 6 shows parameter estimates from fitted regression models that included comorbidities and age and sex for total healthcare costs. Multiplicative costs (cost ratios) were reported for each comorbidity along with marginal differences calculated on the dollars scale (for the reference patient).

**Table 6:** Total healthcare costs: Estimated cost ratios and marginal differences in costs.

	EDS adult cohort			EDS child cohort		
Parameter	Cost ratio (95% CI)	*p*-value	Marginal cost difference*	Cost ratio (95% CI)	*p*-value	Marginal cost difference*
Dysautonomia (POTS)	1.29 (1.14–1.45)	<0.001	$3,400	1.47 (1.10–1.96)	0.009	$3,400
Mast cell disorders	1.35 (1.10–1.64)	0.003	$4,100	1.40 (0.78–2.50)	0.260	$2,900
Gastrointestinal	2.04 (1.66–2.51)	<0.001	$12,400	2.37 (1.36–4.13)	0.002	$10,000
Depression/anxiety	1.22 (1.09–1.37)	<0.001	$2,700	1.60 (1.24–2.05)	<0.001	$4,400
Musculoskeletal	1.31 (1.15–1.49)	<0.001	$3,700	1.46 (1.08–1.97)	0.013	$3,400
Neurological	1.42 (1.26–1.60)	<0.001	$5,000	1.51 (1.13–2.02)	0.006	$3,700
Other	1.33 (1.19–1.49)	<0.001	$3,900	1.54 (1.17–2.04)	0.002	$3,900
Age, 10-year increase	1.06 (1.02–1.11)	0.006	$700	1.07 (0.72–1.59)	0.73	$2,100
Sex (male vs. female)	1.13 (0.96–1.34)	0.150	$1,600	1.01 (0.78–1.30)	0.95	$60
CCI score, 1-point increase	1.16 (1.13–1.19)	<0.001	$1,900	1.29 (1.17–1.43)	<0.001	$2,100
Dysautonomia (POTS)	1.20 (1.05–1.36)	0.007	$2,100	1.28 (0.93–1.75)	0.12	$1,700
Mast cell disorders	1.24 (0.91–1.69)	0.18	$2,600	1.58 (0.59–4.24)	0.36	$3,500
Gastrointestinal	1.36 (1.02–1.82)	0.035	$4,000	1.58 (0.66–3.81)	0.31	$3,500
Depression/anxiety	1.38 (1.25–1.52)	<0.001	$4,100	1.78 (1.43–2.23)	<0.001	$4,700
Musculoskeletal	1.27 (1.13–1.43)	<0.001	$2,900	1.60 (1.24–2.06)	<0.001	$3,600
Neurological	1.31 (1.16–1.48)	<0.001	$3,400	1.54 (1.17–2.02)	0.002	$3,200
Other	1.43 (1.29–1.58)	<0.001	$4,600	1.93 (1.47–2.52)	<0.001	$5,600
Age, 10-year increase	1.05 (1.01–1.09)	0.012	$500	1.05 (0.77–1.43)	0.77	-$300
Sex (male vs. female)	1.12 (0.97–1.30)	0.11	$1,300	1.08 (0.87–1.34)	0.49	$500
CCI score, 1-point increase	1.15 (1.12–1.18)	<0.001	$1,600	1.31 (1.20–1.43)	<0.001	$1,900

*Marginal cost difference was estimated after setting all other comorbidities to not present, age to 34, and sex to female (reference patient).

As reported in Table 5, for the EDS and HSD cohorts, both adults and children, with any of the comorbidities had greater healthcare costs. The largest difference was found in the EDS cohorts with gastrointestinal comorbid conditions, with more than double the costs (Adults: CR = 2.04; Children CR = 2.37), which reflected a marginal cost increase of $12,400 for the reference adult patient and $10,000 for the reference child patient. Patients with HSD, both adults and children, showed similar patterns, but to a lesser degree. Age and sex had less impact on marginal costs, however, differences in the comorbidity index were significant in all cohorts, adding to costs.

### 3.6 Estimates of excess cost

The estimated excess costs for patients with EDS and HSD are shown in Table 7. In 2021, adults with EDS and HSD had total healthcare commercial insurance costs that were $21,100 and $11,600 more than the matched comparison groups without EDS and HSD. Children with EDS and HSD had total healthcare commercial insurance costs that were $17,000 and $11,000 more than the matched comparison groups without EDS and HSD.

**Table 7:** Estimated excess costs for patients with EDS and HSD.

	Cohort	Total healthcare costs Mean diff (95% CI)
Adult	EDS cohort	$21,100 ($19,400–$22,900)
	HSD cohort	$11,600 ($10,300–$12,900)
Child	EDS cohort	$17,000 ($14,300–$19,900)
	HSD cohort	$11,000 ($9,500–$12,700)

## 4 Discussion

This research study is the first to examine the cost burden of EDS and HSD in United States. We found that patients in the MarketScan database, adults and children, who had EDS or HSD had substantially higher associated excess healthcare costs than patients without EDS or HSD when considering age, sex, geographic location, and comorbidities. Because other variables not included in the MarketScan database, such as socioeconomic factors, may also influence excess costs, we cannot conclude that EDS/HSD is the sole causal reason for these large excess costs.

A wide spectrum of diseases and disorders have been associated with EDS (14). Functional gastrointestinal disorders (disorders of the gut-brain interaction) are widespread (20–23). Autonomic dysfunction, specifically postural orthostatic tachycardia syndrome (POTS) and orthostatic hypotension are well-documented (16–19). More recently, potential associations with mast cell disorders are documented (24–27). Anxiety and depression are also well-documented (28–31). We examined the most frequently occurring comorbidities that are likely to impact costs in this population. The findings support the assumption that EDS/HSD specific comorbid conditions contribute to overall costs.

While the literature says that HSD is of comparable severity to hEDS (32) our data indicate that those diagnosed with hypermobility syndrome are incurring lower healthcare costs and thus are likely not as severe in terms of their clinical presentation. As expected, costs were higher for patients with vascular type EDS because of their need for emergency care likely due to catastrophic events (e.g., vessel rupture, arterial dissection).

The overall prevalence of EDS has been estimated to be 1 in 5000 (28, 33, 34), however, scientific evidence is lacking to support this number and the prevalence of EDS differs for each of the 13 types. Classical EDS has a prevalence of 1 in 20,000–40,000. Vascular EDS has a prevalence of 1 in 100,000–200,000 (1). Hypermobile type EDS (hEDS) is the most common, but the exact prevalence has been difficult to estimate due to changes in categorization. A recent study reports a combined prevalence of hEDS and joint hypermobility syndrome (JSD) in Wales of 1 in 500 (3). Since misdiagnosis is common, the actual number may be much higher. It is important to highlight that the costs reported in our results are only from those with an established diagnosis. The average time from the onset of symptoms to a diagnosis in hEDS can be over a decade (4). Previous research shows if undiagnosed, chronic illnesses can have even higher costs (13). These conditions have the hallmarks of rare disease including lack of research, few treatments under development, little funding, lack of public awareness, and dependence on patients’ groups for advocacy (35).

### 4.1 Limitations

This study has several limitations. Selection bias is a limitation of our study. MarketScan is a very large convenience sample, but we cannot say that it is nationally representative because there may be differences in patient characteristics and healthcare plans that differ from the universe of all privately insured individuals. This to our knowledge has not been assessed. All of the study cohort patients had EDS/HSD, defined as at least one outpatient claim in 2021. We do not know when patients were diagnosed. Socioeconomic variables that impact having commercial insurance coverage are not included.

Notably, most EDS patients (79.5%) in this dataset were assigned the ICD-10 code “Unspecified” (Q79.60). This limited our ability to analyze subtypes. The large percentage coded unspecified could be explained by patients with overlapping types, uncertainty about types, or one of the rare types that do not have an ICD-10 subcode. Likewise, the study selection criteria for the EDS/HSD comorbid conditions were dependent on the selection of ICD-10 codes. We do not know the year that the patient was originally diagnosed; thus, we could not determine whether costs differed for patients who were newly diagnosed compared to those receiving an established treatment course. Another limitation is that there are no published diagnostic criteria for hypermobility syndrome (M35.7) and no diagnostic codes for HSD. Finally, our findings may not be applicable to patients with noncommercial insurance.

## 5 Conclusion

Patients living with EDS and HSD have high unmet needs, with high biopsychosocial and economic impacts. This burden affects patients, families, and health systems. Our study found that patients diagnosed with EDS or HSD had significantly greater healthcare costs than patients without EDS or HSD. Disproportionate healthcare costs in this population have health policy and economic implications. The drivers of disproportionate healthcare costs need to be understood. For example, does earlier diagnosis and appropriate referrals to specialist care, when necessary, bring down overall costs? If diagnosed and treated early and effectively, costs for EDS patients may be reduced and more predictable. It is our hope that this study highlighting the excess costs of EDS/HSD will raise interest and recognition of EDS/HSD as a public health problem. Policy implications include timely diagnosis, access to optimal treatment, and accelerated research to improve treatments.

## Data availability statement

The datasets presented in this article are not readily available because the dataset analyzed for this study was obtained by Penn State College of Medicine from Merative and is subject to restrictions under the license agreement. Requests to access the datasets should be directed to jschubart@psu.edu.

## Ethics statement

Ethical review and approval was not required for the study on human participants in accordance with the local legislation and institutional requirements. Written informed consent from the patients/participants or patients/participants' legal guardian/next of kin was not required to participate in this study in accordance with the national legislation and the institutional requirements.

## Author contributions

JS: Conceptualization, Investigation, Methodology, Project administration, Supervision, Writing – original draft, Writing – review & editing. ES: Data curation, Formal analysis, Methodology, Writing – original draft, Writing – review & editing, Conceptualization. DK: Writing – original draft, Writing – review & editing, Investigation. SM: Writing – original draft, Writing – review & editing, Investigation. CF: Writing – original draft, Writing – review & editing, Conceptualization, Investigation.
